# Effect of different garments on thermophysiological and psychological comfort properties of athletes in a wear trial test

**DOI:** 10.1038/s41598-023-42085-2

**Published:** 2023-09-09

**Authors:** Esra Taştan Özkan, Binnaz Kaplangiray, Ufuk Şekir, Şenay Şahin

**Affiliations:** 1 Traditional Turkish Arts Department, Bitlis Eren University, Merkez, Bitlis Turkey; 2 Textile Engineering Department, Uludağ University, Nilüfer, Bursa Turkey; 3 Sport Medicine Department, Uludağ University, Nilüfer, Bursa Turkey; 4 Coaching Education Department, Uludag University, Nilüfer, Bursa Turkey

**Keywords:** Physiology, Engineering, Materials science

## Abstract

This paper reports on an experimental investigation of thermophysiological and psychological responses during and after an incremental low- to high-intensity exercise at 27 °C and 45% humidity. Five t-shirt garments were produced from different yarn types, their weights and yarn counts were close to each other. During the wear trials, heat and humidity sensors were placed at four body locations (the chest, back, abdomen, and waist). In addition, dynamic comfort measurements of the upper body were examined using a datalogger and subjective rating scales. This study aimed to investigate the effects of garment type on aerobic performance, microclimate temperature and humidity values, and psychological comfort. It was observed that the relative humidity and temperature of the microclimate were low in fabrics with high air permeability and low thermal resistance values of the Tencel single jersey and polyester mesh knitted fabrics. There was a significant difference in microclimate temperature results of TS coded Tencel single jersey t-shirt sample and other t-shirt samples according to statistical analysis results. On the other hand, the statistical results of the PM coded fabric sample measured at lower humidity in the three body regions were found to be a significantly different from those of the other samples (except TS). Although not statistically significant, the VO2 values and heart rates of these fabrics were lower than those of other fabrics. It was concluded that garments made from Tencel single jersey (TS) and polyester mesh (PM) fabrics affected the performance of athletes positively. Athletes were less forced during the training, and the activity could be maintained more than the others when wearing these clothes.

## Introduction

Athletes need comfortable clothes to continue the motion during high intensity ativities. Therefore, sports clothes must keep the wearer cool and dry. During high-intensity exercise, athletes sweat and lose excess heat and humidity from the microclimate. A microclimate is defined as the air layer between the skin and clothing, from which heat and vapour transfer occur. During sweating, the moisture transfer rate of clothing is slow, and the relative and absolute humidity levels of the clothing microclimate increase, thereby suppressing the evaporation of sweat. This increases rectal and skin temperatures, and resulting in heat stress^1^. Thermophysiological comfort is related to the thermal balance of the human body, which maintains an internal body temperature of approximately 37 °C^2^. Cooling of the human body by sweat evaporation is the only natural means to remove heat from the body in conditions of a hot environment and to maintain the clothing wearer in conditions of thermal comfort.

The heat and moisture transfer properties of clothing also affect the performance of athletes. Clothing that makes an athlete feel wet and warmer negatively affects the maximum oxygen consumption capacity (VO2max value), causing the athlete to feel tired in a shorter time. The following studies were conducted on subjective wear trials.

Two different studies investigated the effects of clothing openings and ventilative designs, such as the mesh, on thermophysiological comfort. The design of pit zipper openings at both the arm and side seams had an effect on thermal regulation and limited the rate of temperature rise. However, ventilative clothing designs, such as placing mesh fabrics in clothing systems, can influence the heat and moisture transfer performance. In addition, skin temperature, clothing microclimate temperature, oxygen uptake, respiratory exchange ratio, sweat efficiency, and comfort sensations were significantly different between mesh fabrics and control piece t-shirts^3^.

Examining the thermophysiological and psychological effects of aerobic clothing, in a study in which body temperature and relative humidity were measured using sensors placed on six different body regions. It was observed that body temperature and relative humidity changed with different trends according to time, clothing type and body region, showing remarkable changes at different times^4^. In studies where specially designed fabrics and clothes (such as promoting sweat evaporation fabric and improved evaporative and ventilation properties, moisture wicking t-shirts) were used in wear trials. The results showed that the fabric type and construction had no effect on psychological, thermoregulation, and comfort sensations. In contrast, the tested t-shirt exhibited superior evaporative characteristics and lower regain quality^5^. In a study investigating the effect of cotton and polyester t-shirts on physiological and psychological thermal responses during exercise and recovery, it was found that PES t-shirts allowed the skin temperature to return to the pre-exercise level faster, and thermal sensation improved, but did not improve any thermophysiological and subjective sensory responses^6^. On the other hand, the thermal-wet comfort of ten t-shirts made of ten types of hygroscopic fibers, such as cotton, wool, lyocell, modal, soybean, bamboo, and their mixtures was investigated, and it was observed that the thermal-wet comfort changed with fiber type^7^. In studies on tracksuits and outdoor jackets, athletes prioritized comfort and lightness when purchasing clothes, and a higher WVP was shown to benefit moisture management during submaximal exercise in a cold environment by reducing the relative humidity in the clothing system^8,9^. The relationship between the subjective evaluation of wearing comfort in a warm environment and objectively determined physiological parameters with five different raw materials was investigated and it was found that the subjective and objective results were related^10^. Skin temperature and heart rate were measured in a study comparing generally used men's work clothes and clothes in which phase change materials are used in work clothes. It has been observed that the use of PCM slightly increases the temperature at low temperatures and slightly lowers the temperature at high temperatures^11^. In another study, the thermal comfort properties of wool and wool blended workwear were evaluated using subjective evaluation methods, and it was observed that the perception of comfort depended on activity level, environmental conditions, and clothing^12^. The tactile comfort, fit, and aesthetic properties of commercially available cycling clothing in a wear trial were investigated, and it was found that tactile comfort and thermal comfort were perceived differently by subjects^13^. Stojanovic et al.^14^ developed a smart t-shirt that contained temperature and humidity sensors to monitor the thermal situation of athletes. Higher temperature values were measured on the subject’s back at the selected ambient temperatures^14^. The effects of wetted inner clothing on thermal strain in young and older males were investigated using a wear trial test when wearing a ventilative jacket. The results showed that wearing a water-soaked inner T-shirt while using a ventilation jacket reduced thermal and cardiovascular strains in young and old individuals working under hot conditions^15^. In another study, sports t-shirts made from cotton and polyester were used in a wear trial, and the skin temperature, heart rate, body sweat loss and sweat absorption of garments were investigated. It was found that wearers preferred smooth, lightweight, and breathable fabrics under neutral environmental conditions^16^.

The objective of the current study was to expose the t-shirt samples to a submaximal exercise test conducted by athletes with a specific rate of oxygen consumption value. This allowed for the assessment of various fibers’ effects on athletes’ performace. While previous studies have mostly focused on cotton and polyester single jersey, different fibers and mesh structure were used in this study. In addition, the effects of fiber differences on the microclimate temperature and relative humidity values of four distinct body locations were investigated using a wear trial test. The best fiber type and garment for athlete performance during high intensity activities, especially for long distance runners were determined. Because the oxygen consumption value was high, the athletes became more tired when that garment was worn.

## Material and method

The properties of the fabrics measured using standard methods are listed in Table 1. Ten healthy male athletes (age: 21 ± 3 years, height: 180.3 ± 5 cm, body weight: 70 ± 5 kg) from Uludag University Sports Science Faculty were chosen to perform the wear trials. All subjects were non-smokers and healthy long distance runners with no history of cardiopulmonary diseases. Each participant read and signed a written informed consent form, consistent with the principles outlined in the Declaration of Helsinki, prior to participation in the study. This study was approved by the Ethics Committee of Bursa Uludag University.

**Table 1:** Fabric properties and clothing insulation value of tested garments.

Fabric code	Composition (%) and yarn count	Knit type	Mass/unit area (g/m^2^)	Thickness (mm)	Air permeability (dm^3^/sn)	Thermal resistance (m^2^ K/W)	Water vapour resistance (m^2^ Pa/W)
CS	100% cotton 19.68 Tex	Single Jersey	150	0.61	1.6	0.0172	2.9
CPS	50–50% cotton/polyester 19.68 Tex	Single Jersey	148	0.57	2.02	0.0187	2.39
POS	91% textured polyester 9% Spandex 11.11/108 Tex textured polyester, 2.22 Tex Spandex	Single Jersey	135	0.48	1.26	0.0112	1.27
PM	98% textured polyester 2% polyester trilobal 24.44/360 Tex textured polyester, 2.22 Tex polyester trilobal	Mesh	146	0.62	3.4	0.0124	1.35
TS	100% Tencel 19.68 Tex	Single Jersey	145	0.52	3.65	0.0097	1.87

**Figure 1:**
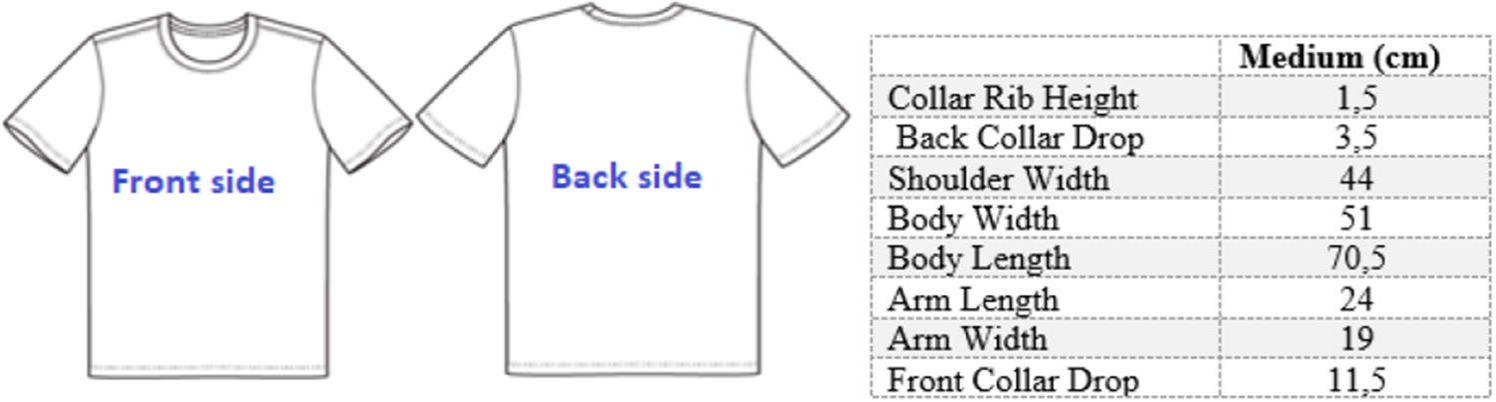
Front and back side of T-shirt samples and garment size.

The thicknesses of the fabrics were measured according to ASTM D1777 standard test method for thickness of textile materials using James H. Heal R&B cloth thickness tester. The air permeability properties of the fabrics were measured using an SDL Atlas air permeability instrument according to the EN ISO 9237 standard determination of the permeability of fabrics to air, with a 100 Pa air pressure and a 20 mm^2^ test area (EN ISO 9237 Standard). The thermal resistance and water vapour resistance of fabrics were measured in a previous study used for comparing the garments^17^. The thermal resistance and water vapour resistance properties of the fabrics were measured using an SDL Atlas Sweating Hot Plate instrument according to the EN 31092 and ISO 11092 standards with a plate surface temperature of 35 °C. Thermal resistance measurements were collected in a controlled room at a temperature of 20 °C and a humidity of 65%. The water vapour resistance measurements were collected at a temperature of 20 °C and humidity of 40%. Five kinds of short sleeve T-shirts were tailored for use in experiments on these fabrics. All fabrics were knitted using the same design and medium size. The design of the T-shirts is shown in Fig. 1.

Each participant visited the laboratory of the Sports Medicine Department on six separate occasions. During the first visit, body weight and height were measured and each participant performed a maximal graded exercise test on a treadmill. In order to assess the maximal aerobic capacity of the participants, an exercise test protocol was employed. This protocol involved conducting a treadmill exercise test to determine maximum aerobic capacity and maximum oxygen consumption values. The test duration averaged 12–15 min on the treadmill, during which the running speed was incrementally increased every 3 min. At the end of this test, the maximal oxygen consumption value (VO2max) for each participant was calculated. During the measurement, the oxygen consumption values of the subjects were analyzed with a metabolic measuring device from the expiratory air by taking the average values for each breath (breath-by-breath) at 30-s intervals. The oxygen consumption value attained at the point of reaching the maximum heart rate was reached (220-years), when the VE/VO2 value reached 30 l/min, or when the subject could no longer sustain the exercise due to its difficulty, was considered as the maximal aerobic capacity (VO2max). VO2max was defined as the maximum capacity of oxygen consumption achieved during exercise. On the following 5 days, the athletes participated in an incremental low- to high-intensity submaximal aerobic exercise. Athletes with a VO2max = 59–64 ml/kg/min value were included in this study and performed the submaximal aerobic exercise. An activity that reaches up to a maximum of 70–80% of the aerobic capacity is defined as submaximal activity. The athletes performed the submaximal exercises at the same time of day and after 3 days of relaxation. Each participant performed the submaximal exercise five times in total at intervals of 4–7 days, wearing four different testing clothes and a control garment at 27 °C and 45% humidity laboratory contions on a treadmill. Participants wore the same shoes, socks, and underwear during each trial. On the morning of each test, the participants were instructed to consume 1 L of water 2 h before the start of the test to ensure proper hydration.

CS coded t-shirt made from cotton single jersey fabric was chosen as the control garment. At the beginning of the test, the participants wore the t-shirts and rested for 10 min in the laboratory conditions to acclimate to the experimental environment. Subjective thermal, wetness, and comfort sensations were recorded during this period. Afterward, the participants performed a wear trial protocol (submaximal aerobic exercise), which consisted of 50 min of followed by 15 min of relaxation. The exercise started with 9.5 km/h running speed at a 1° slope and the speed was increased every ten minutes. The exercise finished with 13.5 km/h speed on a 1° slope. The relaxation period consisted of 5 min of 5 km/h speed at 0° slope and 15 min of sitting on a chair. Measurements were taken immediately before, during, and after the submaximal exercise.

**Figure 2:**
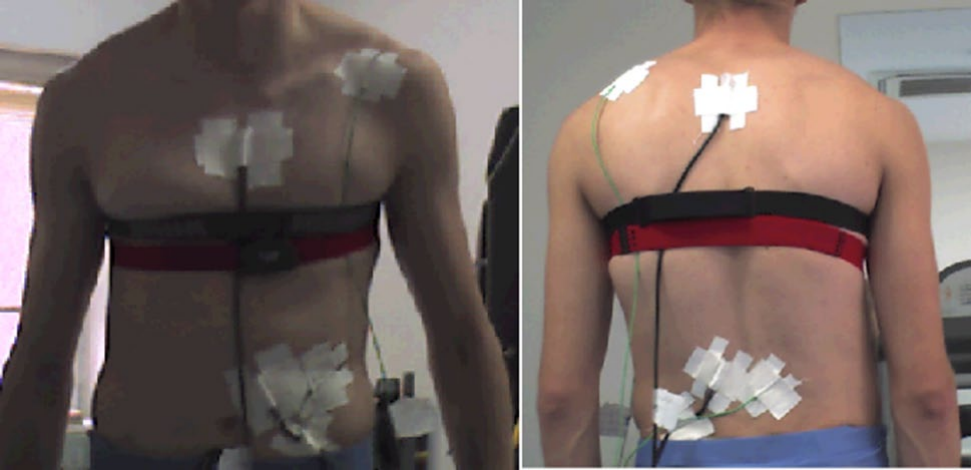
Position of sensors on four different body locations.

A datalogger (Almemo, Germany) was used in the experiments to measure the microclimate temperature and relative humidity from four different body locations (chest, back, abdomen, and waist) during all exercise protocol. The sensors are shown in Fig. 2. In addition, subjective evaluation scales were used to measure the subjective thermal, wetness, and comfort sensations. Five point scales were used for wetness and comfort sensation and 7 point scale was used for subjective thermal sensation (Table 2). The rating of perception was reported by the subjects at each scheduled time before, during, and after exercise.

**Table 2:** Five and seven point scales for subjective sensations.

1	Cold	1	Dry	1	Comfort
2	Cool	2	Slightly damp	2	Less comfortable
3	Neutral	3	Damp	3	Uncomfortable
4	Slightly warm	4	Wet	4	Very uncomfortable
5	Warm	5	Very wet	5	Very very uncomfortable
6	Hot				
7	Very hot				

Before and after applying the wear trial test protocol, participants were weighed with and without garments. Body sweat loss and the amount of sweat absorbed by t-shirt samples were calculated. Furthermore, cardiovascular responses (HR) were measured during and after exercise using a heart rate monitor (Polar). A Polar V800 HRM with a Polar H7 Heart Rate Sensor chest strap (Henceforth, V800) recorded RR intervals at a sampling frequency of 1000 Hz^18^. The maximum oxygen consumption of the athletes was measured with a Fitmate™, which is a new small (20 × 24) metabolic analyzer (Cosmed, Italy). FitMate™ uses standard metabolic formulas to calculate oxygen uptake, and energy expenditure is calculated using a fixed respiratory quotient (RQ) of 0.85^19^.

## Results

### Physiological variables

**Figure 3:**
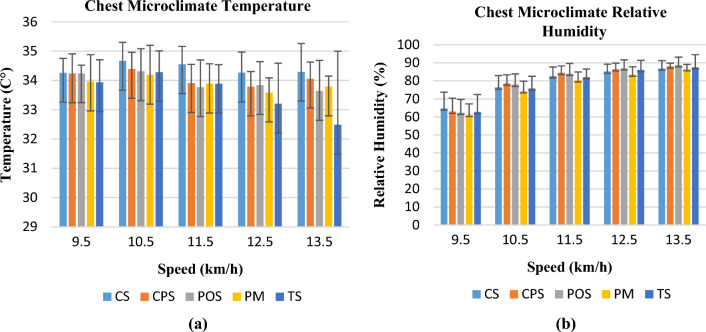
Temperature (**a**) and relative humidity change (**b**) in the chest region.

Datalogger measurements of microclimate temperature and relative humidity for the four body regions are examined in this section. Changes in chest microclimate temperature and relative humidity with activity speed are shown in Fig. 3a,b. The highest chest microclimate temperatures were observed in the highest thermal resistance values CS cotton single jersey and CPS cotton polyester fabrics. In a previous study, polyester and cotton surface temperatures were compared at 24 °C during exercise and rest, and sudden increases in the cotton clothing surface temperatures were observed during exercise. This is because cotton absorbs moisture and releases a certain amount of heat, which may be responsible for the high surface temperature of the garment^20^. The lowest temperatures were measured during and at the end of the activity in the TS Tencel single jersey T-shirt, which has the highest air permeability and the lowest thermal resistance value.

The highest relative humidity value at the beginning of the activity was observed in the CS coded sample, which also exhibited the highest water vapour resistance value. Lower relative humidity values were observed in one of the lowest water vapour resistance and second highest air permeability value PM polyester mesh knitted fabric. Similar to a previous study, water vapour diffusion was mainly dependent on the porosity of the fabrics^21^. The chest microclimate temperature varied between 32 and 35 °C. The highest temperature values were reached when the speed was 10.5 km/h, and the lowest temperature value was reached when the speed was 12.5 km/h which sweating occurs at this rate. For all garment samples, the relative humidity value increased with increasing speed (starting at 60% and reaching approximately 90%). The greatest increase in the relative humidity value occurred in the speed range of 9.5–10.5 km/h, where it was measured at the highest temperature in the clothes. In the 10.5–11.5 speed range, where the humidity increase rate decreased, the temperature values of the clothes also decreased owing to perspiration.

**Figure 4:**
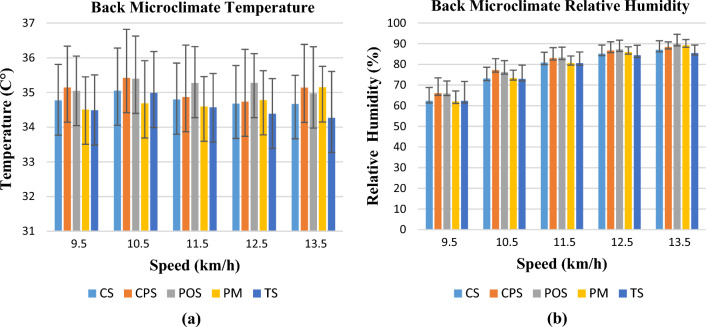
Temperature (**a**) and relative humidity change (**b**) in the back region.

Changes in the back microclimate temperatures and relative humidity at different activity speeds are shown in Fig. 4a,b. Similar to the chest microclimate region, the highest air permeability and the lowest thermal resistance value TS coded Tencel single jersey knitted fabric showed the lowest microclimate temperature values throughout the activity and at the end of the activity. The highest back microclimate temperature values were measured for the POS polyester single jersey knitted fabric, which had the lowest air permeability value. The lowest back microclimate relative humidity values were observed in the CS coded cotton and TS coded Tencel single jersey fabrics. Similar to the results of this study, in a previous study, cotton fabric absorbed more moisture in the back area and reduced the microclimate relative humidity value^22^.

Unlike the chest microclimate region temperature, the lowest temperature values were recorded in the speed range 11.5 km/h. This means that sweating in the back region begins before sweating in the chest region. Similar to previous studies, the back microclimate temperatures were higher than the chest microclimate temperatures throughout the activity (34.5–35.5 °C)^23^. The lowest temperature values were observed in the speed range 11.5 km/h. The back microclimate relative humidity values were in the same range as those of the chest microclimate relative humidity values (Fig. 4b).

**Figure 5:**
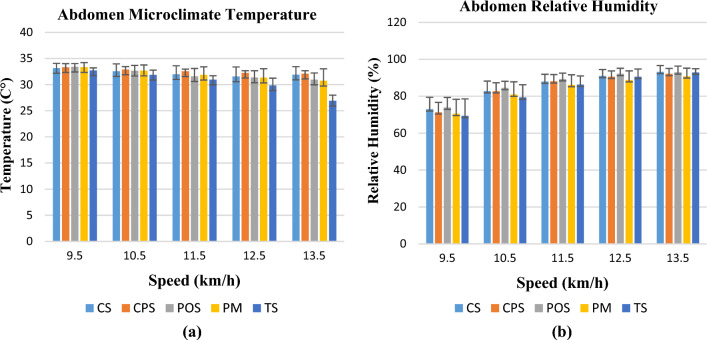
Temperature (**a**) and relative humidity change (**b**) in the abdomen region.

Changes in abdomen microclimate temperatures and relative humidity at different activity speeds are shown in Fig. 5a,b. The highest microclimate temperature value was measured at the highest thermal resistance value of the CPS coded cotton polyester garment during the activity. The lowest miroclimate relative humidity value was seen as one of the lowest water vapour resistance PM coded polyester mesh knitted sample at the end of the activity. If the water vapour transfer rate of the garment is low, the relative humidity in the microclimate increases, making it more difficult to throw out the sweat from the microclimate^2^. Although the TS coded Tencel fabric had the highest air permeability value, its microclimate relative humidity value was higher than that of the fabric with the mesh knit structure. This is most probably due to the porous nature of the mesh structure; liquid sweat can be discharged to the outside more easily. From this point of view, among the examined T-shirts, POS, CS, and CPS are the ones that keep the wearer damper than others.

Abdomen microclimate temperatures were measured lower than the chest and back microclimate temperatures. Unlike the chest and back, the highest microclimate temperatures were observed at the beginning of the activity, and this value decreased with increasing speed throughout the activity. Owing to the basic model of the T-shirt, it did not fit the body in the abdomen and waist regions and allowed air exchange. The relative humidity values of the abdomen were higher than those of the chest and back regions (between 70 and 95%).

**Figure 6:**
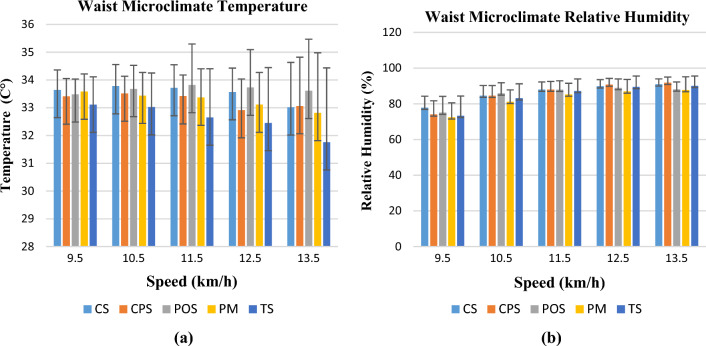
Temperature (**a**) and relative humidity change (**b**) in the waist region.

Changes in waist microclimate temperatures and relative humidity at different activity speeds are shown in Fig. 6a,b. Similar to the other three body regions, the lowest thermal resistance value TS coded Tencel single jersey knitted sample showed the lowest waist microclimate temperatures. The highest relative humidity value was observed for the highest water vapour resistance value CS coded cotton single jersey fabric at the beginning and end of the activity. As a result, fabrics with high water vapor resistance had difficulty evacuating excess moisture during the wear trial, causing the microclimate relative humidity value to be high. The lowest relative humidity value was observed in the PM coded polyester mesh knitted sample, similar to the other two regions (chest and abdomen). It can be said that the mesh structure of the garment makes the person feel more comfortable by providing excess water vapour to the outer atmosphere.

Statistical analysis of the datalogger measurements was performed using SPSS 23.0 program. A two-factor (fabric type × time) repeated measures analysis of variance (ANOVA) was used to analyze the effect of different fabric types and the changes in time for temperature measurements of different body locations. If there is a difference between variables, post-hoc tests can be used to define the relationships between the parameters. Post-hoc statistical tests provide a consistent and appropriate approach for research. Homogeneous characteristics of variance and equality of sample size characteristics are important factors in the selection of post-hoc tests. Where a significant main effect was found a post-hoc test with Bonferroni correction was applied to locate the difference. For all statistical analyses, p ˂ 0.05 was considered significant. The significance of fabric type (p = 0.000 ˂ 0.05), time (p = 0.000 ˂ 0.05), and fabric type × time (p = 0.617 > 0.05) on microclimate temperature results were measured using ANOVA tests. There was a statistically significant difference between the TS Tencel single jersey knitted fabric and the other four t-shirt samples according to the microclimate temperature values (significance of CS p = 0.000 ˂ 0.05, for CPS p = 0.000 ˂ 0.05, for POS p = 0.02 ˂ 0.05, for PM p = 0.028 ˂ 0.05). The point that draws attention here is that the temperature values of the sensor data in four body regions of this t-shirt sample were measured lower than the other four fabrics. As in the study by Li^24^, significant effects of fiber type on the temperature and humidity values of clothing microclimate were observed.

The humidity values of the tested T-shirt samples were compared using SPSS 23.0, and a two factor (Fabric Type * Time) repeated measures analysis of variance (ANOVA) was used to analyze the effect of different fabric types and the changes in humidity for different body locations. The two-way ANOVA test results showed the significance of fabric type (p = 0.000 ˂ 0.05), time (p = 0.000 ˂ 0.05) and fabric type × time (p = 0.998 > 0.05). When the moisture results were examined, a significant difference was observed between the PM and TS coded samples and the other three samples. Statistical analysis results were obtained in conjunction with the sensor measurement results. Clothing made of PM coded polyester mesh fabric measured at lower humidity in the chest, abdomen, and waist region showed a statistically significant difference from the other samples (significance of CS p = 0.033 ˂ 0.05, for CPS p = 0.003 ˂ 0.05, for POS p = 0.000 ˂ 0.05) except for the TS coded sample (p = 1 > 0.05). In addition, the Post-Hoc Bonferroni test results showed that there was a significant difference between the TS coded fabric and the other two samples (significance of CPS p = 0.035 ˂ 0.05, for POS p = 0.006 ˂ 0.05).

### Metabolic measurements

**Figure 7:**
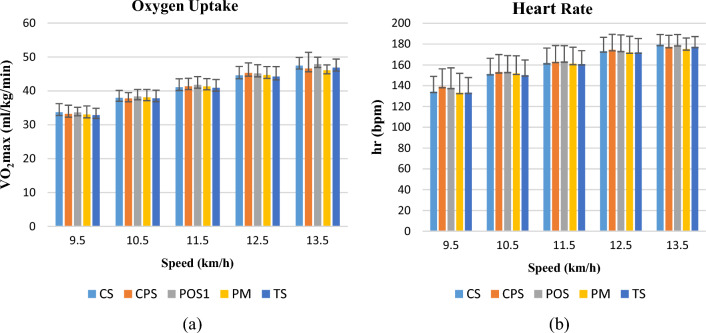
(**a**) Comparison of VO2max values of subjects with speed (**b**) Comparison of heart rates of subjects with speed.

Oxygen uptake (VO_2_) is defined as the amount of oxygen that a person can use per unit of time; the higher this value, the higher the aerobic capacity of the person. Delaying the rise in oxygen uptake during an activity, thus reaching the maximal values (VO2max) later, means that the garment has a positive effect on aerobic performance. The changes in VO_2_ and heart rate with respect to time for the five different types of test garments are shown in Fig. 7a,b. It was seen that VO_2_ values of all garments showed a similar change. At the beginning of the activity, VO_2_ values were 30 (ml/kg/min), which reached 50 (ml/kg) at the end of the activity. It was seen that the heart rate reached about 180 bpm at the end of the activity while it was in the range of 130–140 bpm at the beginning of the activity. TS coded Tencel single jersey knitted sample showed the lowest VO2 value between 9.5 and 12.5 km/h speeds. PM coded polyester mesh fabric exhibited the lowest oxygen uptake value at the end of the activity. When wearing PM and TS garments, the heart rate was generally lower than that of the other garments. In addition, the microclimate temperature and relative humidity of these garments were lower than those of the others, indicating that the increase in microclimate temperature and relative humidity due to the garment increased the heart rate at the same activity level.

### Absorbed sweat rate of garments

**Figure 8:**
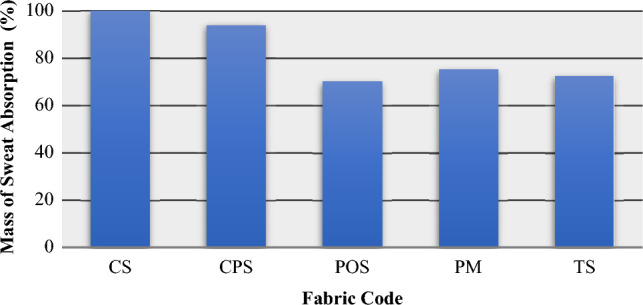
Rate of sweat retention during wear trial test.

Before and after applying the wear trial test protocol, the subjects were weighed with and without a garment. In addition, t-shirt samples were weighed before and after the test and the rate of sweat that the t-shirts held in their structures was calculated. The amount of sweat absorption is shown as a percentages of the t-shirt weight in Fig. 8. The highest rate of sweat retention was observed in the CS cotton single jersey knitted fabric, which was due to the high moisture absorbency of cotton. The second highest percentage of sweat retention was observed in the CPS-coded polyester cotton fabric. It can be said that the T-shirt samples containing the cotton yarn absorbed more amount of sweat. The lowest percentage of sweat retention was observed in the sample made from the POS coded polyester yarn. The comfort properties of clothes made of knitted fabrics with different yarn types (cotton, polyester and blends) investigated and observed that Jacquard polyester T-shirts had lower sweat rates than double-sided polyester and cotton shirts^22^. It has been observed that polyester absorbs sweat, but does not retain it, because of its wicking properties.

### Psychological variables

The subjective thermal, wetness, and comfort sensation values of the subjects before, during, and after the wear trials were measured using a questionnaire. A five point scale was used for wetness and comfort measurements and a seven point scale was used for temperature sensations (Table 2). The responses of the participants to the questions were evaluated according to the average values. The subjective temperature, wetness, and general comfort perceptions of the garments were evaluated using SPSS 23 program. The Kruskal Wallis test, which is a non-parametric equivalent of the ANOVA test, was used to evaluate the subjective evaluation results. With the help of this test, it was tried to determine whether there was a subjectively significant difference between the five garments.

**Figure 9:**
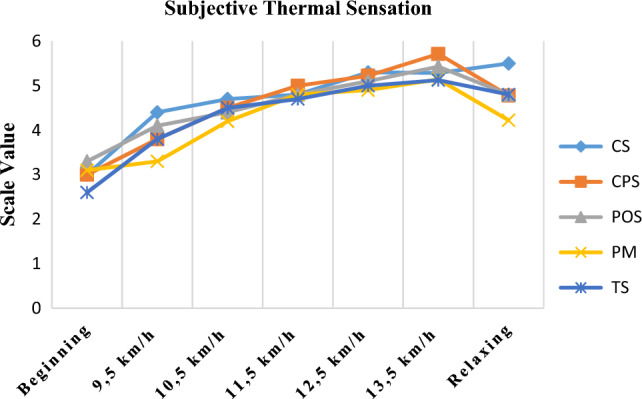
Subjective thermal sensation results of garments.

The subjective thermal sensations of different garments are shown in Fig. 9. At the beginning of the activity, all garments were evaluated as neutral except the TS-coded garment. This fabric was evaluated as cool according to the scale used. In the first part of the activity (9.5 km/h) the thermal sensation was assessed by the subjects as neutral-warm, while in the last part of the activity this value (13.5 km/h) was evaluated in a hot to very hot range. In the rest period, CS coded single jersey knitted garment was evaluated between hot and very hot, while PM coded polyester knitted garment performed between warm-hot scale and felt colder than other garments. In other words, PM and TS coded fabrics with lower microclimate temperatures were also perceived subjectively at lower temperatures. On the other hand, the statistical analysis results showed that there was no significant difference between garment type and subjective temperature evaluation results (Kruskal Wallis = 7.352; p = 0.118 > 0.05).

**Figure 10:**
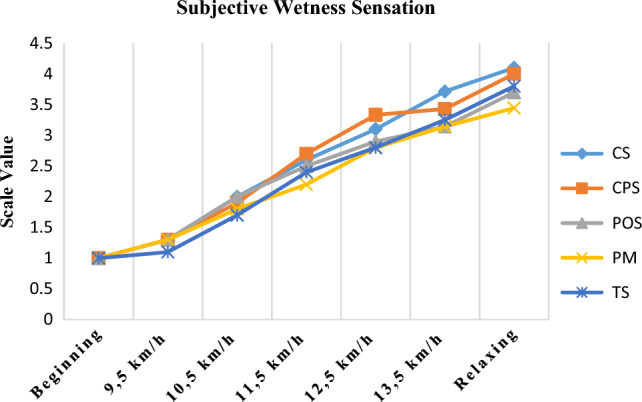
Subjective wetness sensation results of garments.

The subjective wetness sensations of different garments are shown in Fig. 10. It was observed that at the beginning of the activity all clothes were evaluated as dry, and this value was felt in the wet-very wet range at the end of the activity. The overall wettability evaluation results of the PM and TS coded fabrics, which measured the lowest relative humidity values in almost all body regions, were found to be lower than those of the other garment structures. This means that the fabrics felt drier than the others did. On the other hand, the statistical analysis results showed that there was no significant difference between garment type and subjective wetness evaluation results (Kruskal Wallis = 2.351; p = 0.671 > 0.05).

**Figure 11:**
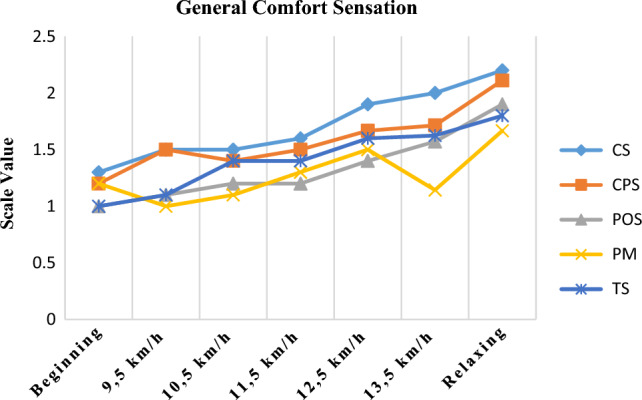
Subjective comfort sensation results of garments.

The subjective comfort sensations of the different garments are shown in Fig. 11. All garment samples were evaluated as comfortable at the beginning of wear trials. However, at the end of the activity (13.5 km/h speed), all the garments were evaluated in the comfort-less comfortable range, except for CS and CPS coded garments. These fabrics were evaluated as uncomfortable at the relaxing time. The important point here was that the wetness values of these garments were higher than those of the other garments during the rest period. The polyester mesh knitted fabric (PM) was evaluated as comfortable by the subjects throughout the exercise. Bertaux et al.^25^ found a significant correlation between the measured temperature and subjective temperature assessment, and between the measured humidity and subjective dampness assessment in a sock wear trial test. There was a significant difference between garment types in terms of general comfort sensations (Kruskal Wallis = 29.551; p = 0.000 < 0.05). There was a statistically significant difference between the cotton polyester single jersey fabric (CPS) and the other four garment types according to Post-Hoc test results (significance of CS p = 0.136 > 0.05, for POS p = 0.013 < 0.05, for PM p = 0.005 < 0.05, for TS p = 0.021 < 0.05). This fabric was measured uncomfortable than other garments according to the results of the statistical analysis. The point that draws attention here is that the objectively measured thermal resistance value of this fabric is higher than other fabrics.

## Discussion

Studies on sportswear are generally based on measurements in the form of fabrics or systems such as thermal manikins. Because the measurements made in the form of fabric are carried out in a static environment, they cannot provide information about the human-clothing interaction in the environment where the clothing is used, and thermal manikin systems have a limited area of use because the tests are very expensive. A wear trial test is defined as a test using human subjects in a specific activity and environmental conditions. Although the variability of some physiological data is high owing to the use of people in wear trials, it is only possible to determine the actual performance of the clothing during use with wear trials. With the help of these tests, the most suitable garment can be selected for that climatic and activity conditions. We explored the thermophysiological and physiological comfort of five different yarn knitted, same model T-shirt samples in a 50-min training program by athletes with a certain oxygen consumption value (VO2max = 59–64 ml/kg/min). We compared the effects of objectively measured thermal resistance, water vapour resistance and air permeability values in the fabric form of garment on the microclimate temperature and microclimate relative humidity values of four body regions obtained from datalogger data. In addition, to compare the subjective evaluations with the data obtained from the datalogger measurements, we asked the subjects to evaluate the garments subjectively with the help of scales during the wear trials. Studies in the literature generally focus on cotton, polyester and their different compositions, a total of five garments which also included a new generation regenerated cellulose (Tencel) were tested in this study. Delaying the increase in oxygen uptake during an activity, thereby reaching maximum values (VO2max) later, means that the suit has a positive effect the aerobic performance. This was an interdisciplinary study, and the effects of clothing on the performance of athletes were investigated by measuring VO2max and heart rhythm values. The result of the statistical tests show that the parameters that affect the microclimate temperature and relative humidity are the fabric type and time. There were no statistically significant effects of regional differences (chest, back, abdomen, and waist) on microclimate temperature and relative humidity characteristics. In terms of garment type, it was observed that the microclimate temperature values of the Tencel fabric were statistically different from those of the other four garments. Similar to the literature, Tencel is suitable for activities performed in hot climates because of the high air permeability and low water vapor resistance values of the fabrics produced with Tencel, which provides a cooler touch even in blends^26,27^.

## Conclusion

The contribution of this study to the literature is to determine the garments that will enable the activities of long distance athletes to continue for a long time. For this purpose, sensors placed in four different parts of the body and subjective scales were used for subjective comfort measurements. In addition, the effects of clothing on VO2max and heart rate were measured during activity. The highest thermal resistance and water vapour resistance value Cotton single jersey (CS) and Cotton-Polyester single jersey (CPS) coded fabrics showed the highest microclimate temperature and relative humidity values in almost all body regions. For garments with a lower water vapour transfer rate, the relative humidity in the microclimate increases, making it more difficult to throw out the sweat from the microclimate. Statistical analyses showed that there was a statistically significant difference between the Tencel single jersey garment (TS) and the other four T-shirt samples according to microclimate temperature values. When the microclimate humidity results were examined, a significant difference was observed between the polyester mesh (PM) and Tencel single jersey (TS) samples and the other three samples according to the Post-hoc Bonferroni test results. Although the subjective temperatures and wetness scale values of the TS and PM coded fabrics were found to be lower than those of the other fabrics, by considering the subjective temperature, wetness, and thermal comfort evaluations there was no statistically significant difference between these fabrics and the others. When the subjective general comfort properties were evaluated, a significant difference was observed between the CPS coded fabric, which had the highest thermal resistance value, and other garments. It was concluded that the objective test results (sensor tests) and subjective evaluation results supported each other. Garments made from Tencel single jersey and polyester mesh fabrics affect the performance of athletes positively, especially considering the results of wear trial tests. In addition, lower VO_2_ and heart rate values were achieved with these garments compared than with the others. It can be concluded that, the athlete is forced less during training and that the activity can be maintained longer when these garments are worn (TS and PM). Although there was no statistical difference between the regions (chest, back, abdomen, and waist) due to microclimate temperature and relative humidity values, it can be said that the temperatures in the back area are higher than those in the chest area, and sweating starts in this area earlier than in the chest area.

## Data avability

All data generated or analysed during this study are included in this published article [and its supplementary information files].
